# Multi-platform molecular profiling of a large cohort of glioblastomas reveals potential therapeutic strategies

**DOI:** 10.18632/oncotarget.7722

**Published:** 2016-02-25

**Authors:** Joanne Xiu, David Piccioni, Tiffany Juarez, Sandeep C. Pingle, Jethro Hu, Jeremy Rudnick, Karen Fink, David B. Spetzler, Todd Maney, Anatole Ghazalpour, Ryan Bender, Zoran Gatalica, Sandeep Reddy, Nader Sanai, Ahmed Idbaih, Michael Glantz, Santosh Kesari

**Affiliations:** ^1^ Caris Life Sciences, Phoenix, AZ, USA; ^2^ Neuro-Oncology Program, Moores Cancer Center, UC San Diego, La Jolla, CA, USA; ^3^ Cedars-Sinai Medical Center, Los Angeles, CA, USA; ^4^ Baylor University Medical Center, Dallas, TX, USA; ^5^ Barrow Neurological Institute, Phoenix, AZ, USA; ^6^ AP-HP, Groupe Hospitalier Pitié-Salpêtrière, Service de Neurologie 2-Mazarin, Paris, France; ^7^ Sorbonne Universités, UPMC Univ Paris 06, UMRS 975, Institut du Cerveau et de la Moelle, Paris, France; ^8^ Inserm U 975, Paris, France; ^9^ CNRS, UMR 7225, Paris, France; ^10^ Pennsylvania State University, Hershey, PA, USA; ^11^ Translational Neuro-Oncology Laboratories, Department of Neurosciences UC San Diego, La Jolla, CA, USA; ^12^ Department of Translational Neuro-Oncology and Neurotherapeutics, John Wayne Cancer Institute at Providence Saint John's Health Center, Santa Monica, CA, USA

**Keywords:** glioblastoma, tumor profiling, EGFRvIII, IDH1, MGMT promoter methylation

## Abstract

Glioblastomas (GBM) are the most aggressive and prevalent form of gliomas with abysmal prognosis and limited treatment options. We analyzed clinically relevant molecular aberrations suggestive of response to therapies in 1035 GBM tumors. Our analysis revealed mutations in 39 genes of 48 tested. IHC revealed expression of PD-L1 in 19% and PD-1 in 46%. MGMT-methylation was seen in 43%, EGFRvIII in 19% and 1p19q co-deletion in 2%. TP53 mutation was associated with concurrent mutations, while IDH1 mutation was associated with MGMT-methylation and TP53 mutation and was mutually exclusive of EGFRvIII mutation. Distinct biomarker profiles were seen in GBM compared with WHO grade III astrocytoma, suggesting different biology and potentially different treatment approaches. Analysis of 17 metachronous paired tumors showed frequent biomarker changes, including MGMT-methylation and EGFR aberrations, indicating the need for a re-biopsy for tumor profiling to direct subsequent therapy. MGMT-methylation, PR and TOPO1 appeared as significant prognostic markers in sub-cohorts of GBM defined by age. The current study represents the largest biomarker study on clinical GBM tumors using multiple technologies to detect gene mutation, amplification, protein expression and promoter methylation. These data will inform planning for future personalized biomarker-based clinical trials and identifying effective treatments based on tumor biomarkers.

## INTRODUCTION

Gliomas are the most common type of primary brain tumors in adults, classified by the World Health Organization (WHO) based on histopathological criteria into four grades: I to IV, with glioblastoma (GBM; grade IV) representing the most frequent and aggressive form [1, 2]. The challenges of GBM treatment include involvement of multiple molecular pathways which result in rapid development of drug resistance, blood-brain barrier considerations as well as molecular heterogeneity of the tumor [3]. The standard-of-care for GBM involves optimal surgical resection followed by a combination of radiation and chemotherapy with the oral DNA alkylating agent temozolomide, which together translates into a median survival of 14.6 months. Almost all GBM patients experience recurrence, and second-line treatments provide only modest benefit for the vast majority of patients. Therefore, there is a desperate need for novel treatment options [4], [5], [6].

Histologically similar GBMs can be driven by distinct genetic events that result in varied clinical behaviors and prognoses. Therefore, effort has been focused on classifying GBMs according to molecular aberrations to better direct therapy [7], [8], [9]. For example, promoter methylation of the MGMT (O6-methylguanine-DNA methyltransferase) gene (MGMT-Me) reduces transcription and, consequently, decreased DNA repair, resulting in enhanced temozolomide sensitivity [10], [11]. Additional actionable alterations including mutations in the active site of isocitrate dehydrogenases (IDH1/2) and EGFR aberrations, e.g. gene amplification and deletion of exon 2-7 (EGFRvIII), have been shown to play an important role in oncogenesis and progression of glioma, and may carry important theranostic significance [7, 12] [13]. TCGA researchers used gene expression-based molecular classification and grouped GBMs into four subgroups of proneural, classical, mesenchymal and neural, as defined by alterations including PDGFRA/IDH1, EGFR and NF1, demonstrating differences in prognosis and responsiveness to aggressive therapies [7]. A comprehensive understanding of the frequencies of these and other important biomarkers in a large cohort of GBM samples from patients would aid clinical trial design and expedite the incorporation of tumor profiling into clinical practice.

Our study aims to investigate biomarker data collected from the molecular profiles obtained in a CLIA-certified laboratory on 1035 clinical GBM tumors to assist in prognostic and therapeutic decisions. Up to 76 biomarkers were selected based on an association with therapeutic responses in clinical studies on various cancer types and tested using multiple technologies. Biomarker results and associated therapies are presented for the full cohort and for subgroups defined by biomarker characteristics such as IDH1 and TP53 mutations. Previous reports have revealed molecular changes during progression of lower grade gliomas to GBM, driving tumor growth and treatment resistance [14]; however such changes during progression of high-grade gliomas have not been systematically reported. Paired tumors available in the database were therefore analyzed for potential biomarker changes over time. Further, we explored the associations of biomarker status with patient survival.

## RESULTS

### Patient and tumor characteristics

**Figure 1:**
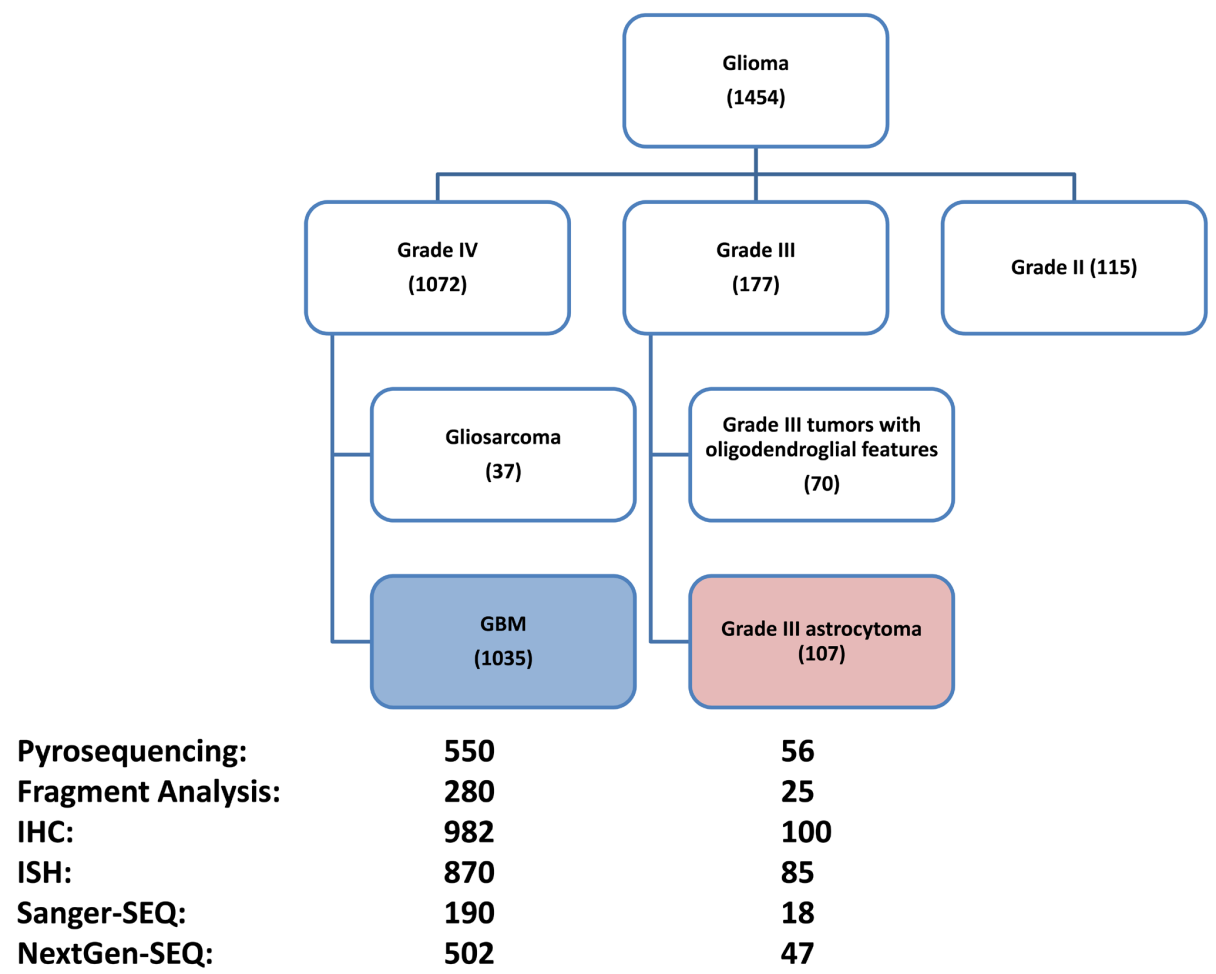
Flow chart showing patient composition included in this study Numbers in parentheses represent the N number of each subgroup. Data analysis was done on the GBM cohort (N=1035); a comparison with grade III astrocytoma (N=107) was also performed. Numbers next to test platforms represent number of tumors with results from each platform. The tests done and test platform used on each tumor are variable, the details of which are shown in Table 1.

A total of 1454 consecutive adult gliomas samples that received tumor profiling from July of 2009 to June of 2015 were identified, from which 177 grade III tumors, 115 grade II tumors 37 grade IV tumors with gliosarcoma features as well as 90 tumors with insufficient tumor grade annotation were excluded. The remaining 1035 GBM tumors were used for biomarker analysis (Figure 1). The average age of the GBM patients were 57.1 years old (interquartile range: 49-66), 413 (40%) were female. From the 177 grade III tumor cohort, 107 tumors of grade III astrocytoma with no indication of oligodendroglial component were used for comparison.

### Distribution frequencies of biomarkers revealed by molecular profiling

A total of 76 biomarkers tested by immunohistochemistry, in-situ hybridization, pyrosequencing, fragment analysis, Sanger sequencing as well as next-generation sequencing were analyzed in the GBM cohort. As different biomarker tests were performed for each tumor, the total N for each biomarker tested varied from 58 to 932 (Tables 1, 2)

**Figure 2:**
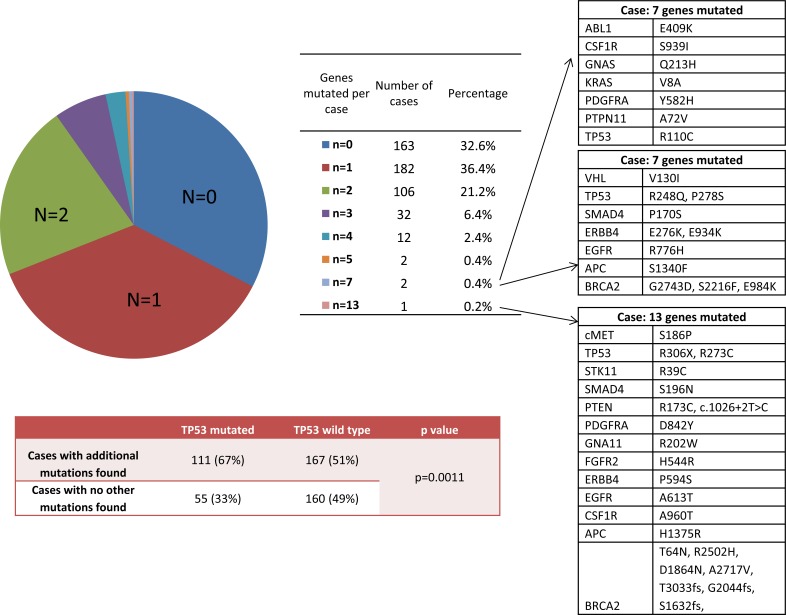
Frequencies of multiple mutations per case (N: number of simultaneous mutations found per case) The 3 cases with the highest number of simultaneous mutations are listed with the specific mutations found. The frequency of TP53 mutation associated with additional mutations is also shown.

Overall, MGMT promoter methylation was seen in 43% of GBM tumors, and EGFRvIII was seen in 19%. In-situ hybridization revealed EGFR amplification occurring in 56%, while 1p19q co-deletion and cMET amplification occurring in less than 2%. Among 22 IHCs, TUBB3 and EGFR overexpression were seen in over 80% of GBM tumors, while intact PTEN expression was seen in 74%. Notably, PD-1 expression on tumor-infiltrating lymphocytes was seen in 46% of GBM tumors using the cutoff of 1/high power field, and PD-L1 expression on cancer cells was seen in 19%, using the cutoff of 5% ([16]). ALK IHC was done on a very small subset of tumors (n=58) and 24% showed overexpression (Table 1).

**Table 1:** Biomarker frequency in GBM tumors tested by pyrosequencing, fragment analysis, *in-situ* hybridization and immunohistochemistry

**Pyro Sequencing**	**Positive (N)**	**Total (N)**	**Percent**
Pyro SEQ-MGMT	235	550	43%
**Fragment Analysis**	**Positive (N)**	**Total (N)**	**Percent**
FA-EGFRvIII	53	280	19%
***In-situ* hybridization**	**Positive (N)**	**Total (N)**	**Percent**
FISH-EGFR	163	289	56%
FISH-1p19q	3	171	1.8%
ISH-cMET	9	500	1.8%
ISH-HER2/Neu	0	609	0
**Immunohistochemistry**	**Positive (N)**	**Total (N)**	**Percent**
TUBB3	401	485	82.7%
EGFR	206	254	81.1%
PTEN	691	932	74.1%
TOP2A	410	786	52.2%
TS	421	819	51.4%
TOPO1	435	847	51.4%
PD-1	112	243	46.1%
ERCC1	166	431	38.5%
RRM1	298	803	37.1%
TLE3	202	601	33.6%
PDGFR	35	129	27.1%
ALK	14	58	24.1%
PD-L1	47	242	19.4%
SPARC	100	733	13.6%
PGP	59	780	7.6%
PR	52	814	6.4%
AR	51	807	6.3%
MGMT	25	434	5.8%
cMET	10	633	1.6%
c-kit	3	298	1.0%
ER	1	818	0.1%
Her2/Neu	0	906	0

List of abbreviations: TUBB3: Class III beta-tubulin; PTEN: phosphatase and tensin homolog; TOPO1: Topoisomerase I; EGFR: epidermal growth factor receptor; TS: Thymidylate synthase; TOP2A: topoisomerase II alpha; RRM1: ribonucleotide reductase subunit M1; ERCC1: excision repair cross-complementation group 1; TLE3: transducing-like enhancer of split 3; PDGFR: platelet-derived growth factor receptor alpha; SPARC: secreted protein acidic and rich in cysteine; PR: progesterone receptor; PGP: P-glycoprotein; MGMT: O-6-methylguanine-DNA methyltransferase; AR: Androgen Receptor; cMET: MET or hepatocyte growth factor receptor; ER: estrogen receptor; Her2/neu: human epidermal growth factor receptor 2; PD-1: programmed death 1; PD-L1: programmed cell death ligand 1; ALK: anaplastic lymphoma kinase; cKIT: CD117 or stem cell factor receptor.

Mutation analysis revealed that 39 of 48 genes tested carried mutations, with frequencies ranging from 0.2% to 34% (Table 2) as calculated from 186-663 samples per gene tested. The highest rates were seen in TP53 (34%), PTEN (16%), EGFR (point mutations and small insertions-deletions) (10%), IDH1 (9%), PIK3CA (8%), BRCA2 (7%) and BRCA1 (5%). The remaining 32 genes showed mutation rates below 5%. Specific protein changes observed in each gene can be found in Supplementary materials.

**Table 2:** Mutation rates of 48 genes tested by sequencing

Sequencing	Positive (N)	Total (N)	Percent	Sequencing	Positive (N)	Total (N)	Percent
TP53	167	494	33.8%	SMO	3	404	0.7%
PTEN	75	460	16.3%	HNF1A	3	433	0.7%
EGFR	50	515	9.7%	ERBB4	3	496	0.6%
IDH1	44	500	8.8%	KDR	3	496	0.6%
PIK3CA	49	596	8.2%	GNA11	2	426	0.5%
BRCA2	12	186	6.5%	FLT3	2	492	0.4%
BRCA1	9	186	4.8%	FGFR2	2	493	0.4%
APC	21	499	4.2%	NOTCH1	2	494	0.4%
ATM	20	490	4.1%	CSF1R	2	496	0.4%
PTPN11	13	494	2.6%	AKT1	2	498	0.4%
JAK3	12	494	2.4%	IDH2	1	309	0.3%
BRAF	16	663	2.4%	SMARCB1	1	497	0.2%
KRAS	13	617	2.1%	MLH1	1	498	0.2%
RB1	10	494	2.0%	CTNNB1	1	499	0.2%
cMET	10	498	2.0%	GNAS	1	500	0.2%
c-KIT	11	566	1.9%	ALK	0	498	0%
PDGFRA	7	491	1.4%	CDH1	0	498	0%
ABL1	6	466	1.3%	ERBB2	0	479	0%
NRAS	6	551	1.1%	FGFR1	0	500	0%
STK11	5	472	1.1%	GNAQ	0	352	0%
VHL	4	422	0.9%	HRAS	0	404	0%
RET	4	478	0.8%	JAK2	0	500	0%
FBXW7	4	495	0.8%	MPL	0	487	0%
SMAD4	4	496	0.8%	NPM1	0	496	0%

While PIK3CA, BRAF, EGFR, cKIT, KRAS and NRAS data are a combination of NextGen and Sanger sequencing, and IDH2 mutation data is from Sanger sequencing, all other mutations were collected using NextGen.

Of the 500 GBM tumors profiled with NGS, 67% had at least one mutation (Figure 2). Co-mutations of 2 genes or more were observed in 31% of patients, 10% had co-mutations of 3 or more genes, and 3 patients showed mutations in 7 or more genes. The genes mutated in these three highly mutated GBM cases are shown in Figure 1. The only common mutated gene among the three cases is TP53. In the complete GBM cohort, TP53-mutated cases were significantly more likely to carry additional mutations in other genes: 67% (111of 166) while only 51% (167 of 327) of TP53-wild type cases had additional mutations (RR=1.31 [1.13-1.52], p=0.0011).

**Figure 3:**
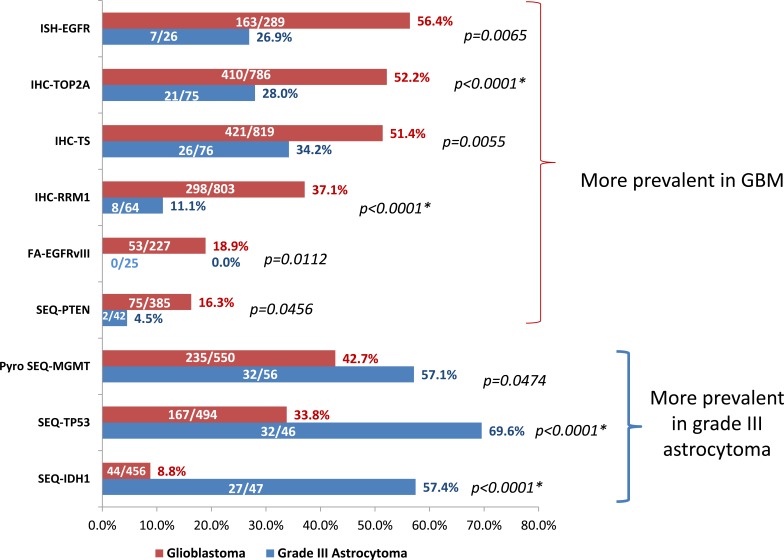
A: Differential biomarker features tested by promoter methylation, fragment analysis, in-situ hybridization and IHC in GBM and grade III astrocytomas Shown are biomarkers that are statistically different in GBM and grade III astrocytomas by two-tailed Fisher-Exact test. Asterisks indicate comparisons that remain statically significant after correcting for multiple comparisons by Bonferroni correction. Numbers on the bar indicates positive N/total N for each biomarker tested.

In order to identify molecular features specific to GBM (WHO grade IV astrocytoma), the GBM cohort was compared to 107 grade III astrocytoma tumors that don't show any indication of oligodendroglial component. As shown in Figure 3, EGFR aberrations including gene amplification and EGFRvIII mutation, PTEN mutation, as well as TOP2A, RRM1 and TS overexpression were significantly more prevalent in GBM than grade III astrocytomas. In contrast, MGMT promoter methylation, TP53 and IDH1 mutations were more frequent in grade III astrocytomas.

### Tumor profiles are differentiated by IDH1 mutation

**Figure 4:**
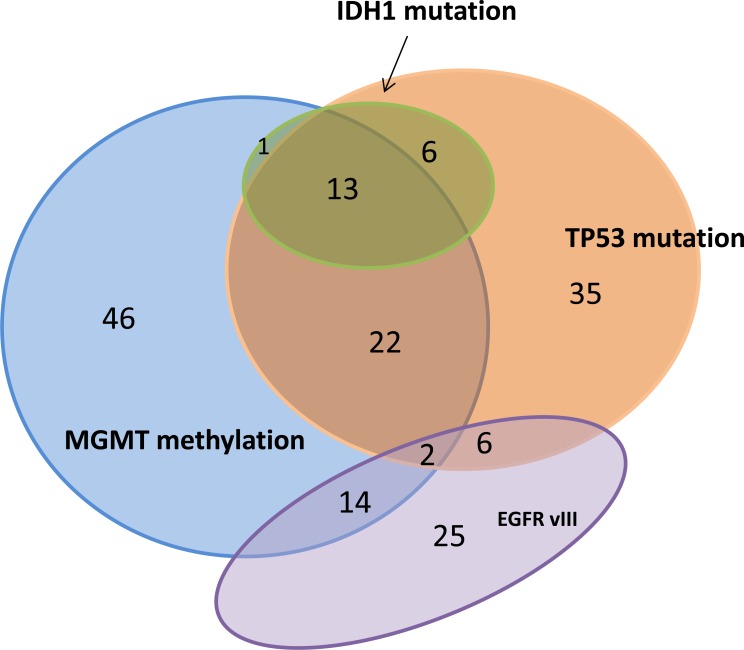
Venn diagram made from 238 GBM cases with IDH1, TP53, MGMT methylation and EGFRvIII evaluated 170 cases showed at least one aberration.

IDH1 mutation identifies GBMs that are developed from lower grade gliomas, i.e., secondary GBM, and are associated with prolonged patient survival [17]. In our GBM cohort, IDH1 mutation was highly associated with MGMT methylation and TP53 mutation. In contrast, IDH1 mutation and EGFRvIII were mutually exclusive, in that all 52 EGFRvIII mutations were found in IDH1 wild type tumors. (Table 3) The relative relationships of IDH1, TP53, MGMT methylation and EGFRvIII are further illustrated in the Venn diagram shown in Figure 4.

**Table 3:** Differential biomarker characteristics in IDH1-mutated and IDH1-wild type GBM

	All GBM tumors			
	IDH1 MT N/Total (%)	IDH1 WT N/Total (%)	RR [95% CI]	*p* value
**MGMT Methylation**	**30/41 (73%)**	**469/423 (40%)**	**3.63 (1.87-7.07)**	<0.0001*
**TP53 mutation**	**40/44 (91%)**	**126/449 (28%)**	**19.7 (7.17-54.1)**	<0.0001*
**EGFR vIII**	0/23 (0%)	52/242 (21%)	0 (n/a)	0.0105
**PTEN mutation**	1/40 (2.5%)	74/420 (18%)	0.13 (0.02-0.94)	0.0117

(Asterisks indicate comparisons that remain statically significant after correction for multiple comparisons.)

Analysis of paired GBM tumor samples reveals biomarker changes over time

**Figure 5:**
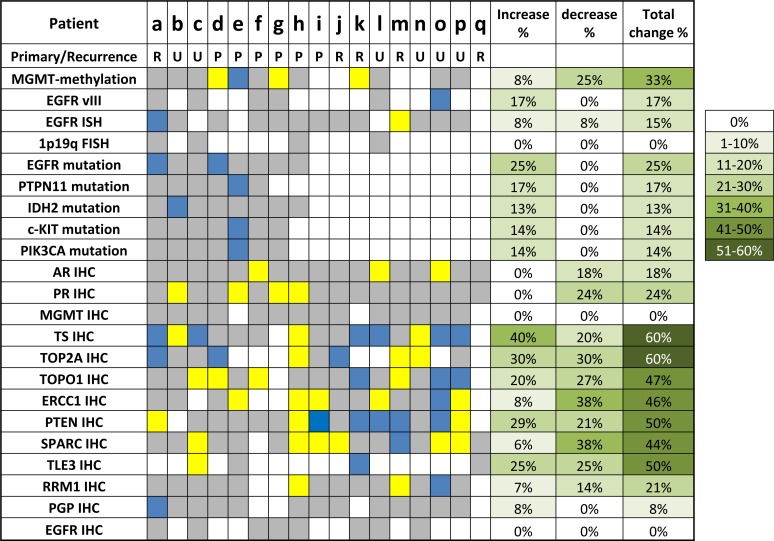
Comparison of biomarker profiles on metachronous GBM pairs (*N*=17) Primary/Recurrence: R, paired recurrent tumors; P: paired primary and recurrent tumors; U: unknown. Yellow: biomarkers that decreased over time, which included loss of protein overexpression by IHC; loss of gene amplification by ISH and loss of gene mutation by sequencing; loss of MGMT promoter methylation by pyrosequencing. Blue: biomarkers that increased over time, which included acquisition of protein expression by IHC, acquisition of gene amplification by ISH; acquisition of gene mutation by sequencing; acquisition of MGMT promoter methylation by pyrosequencing. Gray: no biomarker change over time.

Metachronous paired GBM tumors were available on 17 patients (Figure 5). The mean interval between sample collection times was 499 (91-1605) days. The paired tumors were comprised of primary and recurrent disease (N=6) as well as paired recurrent tumors (N=5), while for 6 pairs it's unclear if the first specimen was from the primary tumor or a recurrence. 94% of the pairs (16 of 17) had at least one biomarker change; patient q only had paired data on 4 biomarkers, and did not show a biomarker change.

Paired MGMT methylation data was available on 12 patients. While 8 tumor pairs had the same methylation status, 3 pairs changed from methylated to unmethylated (Patients d, g and k) and one pair changed from methylation equivocal to methylated (Patient e). (7% methylated to 54%). EGFR gene amplification status changed in 2 out of 13 pairs, with one patient acquiring amplification and one losing amplification (patients a and m, respectively). EGFRvIII changed from absent to present in 1 of 6 pairs tested.

Sequencing data was available on 8 pairs (Patients a-h). While 4 pairs carried the same mutational profiles, 4 pairs acquired new mutations: 2 acquired EGFR mutations (Patient a with EGFR D770_N771insN, Patient d with EGFR T790M), one (Patient b) acquired an IDH2 mutation (P167L) and one (Patient e) acquired three mutations between paired samples collected 4.4 years apart. The first profile of patient e had wild type cKIT, PTPN11 and PIK3CA and only 7% MGMT promoter methylation (equivocal). The second profile showed mutations in cKIT (E583K), PTPN11 (A72T) and PIK3CA (D434N) and 54% MGMT methylation. The same truncating PTEN mutation (R11X) was seen in both profiles. Mutation status of TP53 was unavailable in the first profile and TP53 P177L was seen on the second.

In addition, significant changes were observed for IHC markers. Interestingly, AR (androgen receptor) and PR (progesterone receptor) showed decreased expression in 3 and 4 patients, respectively while no increase were seen. Pgp increased in 1 out of 13 pairs while EGFR and MGMT expression did not change in the 8 and 14 patients with paired data available. No directional patterns were seen in the other markers tested.

**Table 4:** Patient age and selected biomarkers were statistically significantly associated with survival of GBM patients (*N* = 310)

Important prognostic factors	Hazard Ratio	95% Confidence Interval	*P* Value
Age (>70 yrs *vs*. <=70 yrs)	1.75	1.31-2.33	0.00013
**Grade IV patients <=70 years old (*N* = 251)**			
SEQ. MGMT.Me (methylated *vs*. unmethylated)	0.44	0.22-0.87	0.02
IHC. PR (positive *vs*. negative)	0.61	0.4-0.94	0.02
IHC. TOPO1 (positive *vs*. negative)	1.34	0.99-1.8	0.05
**Grade IV patients >70 years old (*N* = 59)**			
IHC. PGP (positive *vs*. negative)	0.31	0.12-0.81	0.02
IHC. PR (positive *vs*. negative)	0.23	0.07-0.8	0.02
IHC. TS (positive *vs*. negative)	2.61	1.11-6.16	0.03

### Tumor grade, patient age and biomarker status were associated with survival

Patient death data was extracted from SSDI by a research intermediate and death data was available for 310 GBM patients. Patient age ranged from 21 to 89 (mean 60) years and mean survival was 543 days. Patients were categorized into the elderly (>70 years old) and young (<=70 years old) based on NCCN treatment stratification. As expected, patients > 70 years old (N=63) had a significantly shorter survival than those <=70 years old (N=247) (HR=1.75, p=0.00013).

Within GBM patients younger than 70 years old, MGMT-Me and positive PR expression were significantly associated with longer survival, and TOPO1 expression was associated with shorter survival. The other biomarkers with data available were not associated with survival. In patients who were older than 70 years old, PR remained associated with a longer survival but MGMT-Me and TOPO1 were not. Instead, PGP expression was associated with longer survival and TS expression with shorter survival. (Supplementary Figures 1 and 2)

## DISCUSSION

Despite tremendous progresses in the molecular characterization of GBM, options for effective treatments are still limited. Clinical trials of targeted therapies and chemotherapies in unselected patient cohorts have shown limited benefit with the exception of temozolomide. There is clearly an unmet need to determine if existing therapies or investigational agents in clinical trials could benefit this population. The use of tumor profiling to guide treatment has generated promising results in various cancers, especially in refractory disease [18] [19] [20] [21]. Even though prospective randomized trials have not been published for all of the biomarker-drug associations utilized in these studies, the improved outcomes observed in patients treated with tumor profiling-guided therapies suggests the potential effectiveness of such an approach. The current study describes a large cohort of GBM tumor samples analyzed with the goal of providing the best treatment options for individual patients (Supplementary Table 3). This approach allowed for identification of potential therapeutic opportunities, both those that are part of standard-of-care and those that are not routinely considered. For example, 43% tumors showed MGMT-Me, suggesting benefit from temozolomide [22] [23] [24]. The presence of EGFRvIII suggests potential utility of EGFRvIII-targeted therapies [25] [26] [27]. Further, agents including gemcitabine and fluoropyrimidines are suggested for a portion of patients based on low RRM1 [28] and TS [29], respectively. While published efficacies of these agents in unselected GBM patients are variable, using predictive markers to select patients who are more likely to respond may increase response rates [30] [31]. Of special interest, tumor expression of PD-L1 and tumor-infiltrating lymphocyte expression of PD-1 are seen in 27% and 48% of tumors, suggesting gliomas as a promising potential tumor type for immune modulatory agents [32]; identification of BRCA1/2 mutations makes potential usage of PARP inhibitors of particular interest [33]; the known high rate of EGFR aberration in GBM was also shown by multiple platforms including IHC, ISH, fragment analysis (for EGFRvIII) and NextGen sequencing (for point mutations and small in-dels), confirming EGFR as an important therapeutic target in GBM [3, 34]. Further investigation into the molecular subgroups of GBM showed that TP53 mutation is indicative of mutations on additional genes and that IDH1-mutated GBM tumors lack EGFRvIII mutations and are more likely to carry TP53 mutation and MGMT promoter methylation. These characteristics of molecular features observed in a large cohort of clinical GBM samples confirm the important role of these genetic events in the genesis of glial tumors and thus the molecular heterogeneity of GBM [9] [35] [36].

We describe here that 94% of metachronous tumor pairs show biomarker changes, potentially resulting from tumor progression and/or treatment-driven selection. Changes in MGMT promoter methylation status have been previously reported with conflicting results [37], [38, 39]. Our observation that 4 of 12 patients experience changes and that loss of methylation is more frequent than acquisition supports the notion that during glioma progression, frequent MGMT promoter methylation changes occur, and therefore patients' responsiveness to temozolomide potentially may also change. Acquired EGFR exon 20 mutations were seen in two patients, one with a T790M mutation and one with an exon 20 insertion (D770_N771insN), both of which are well-studied in NSCLC as an acquired [40] and *de novo* resistance mechanism to EGFR tyrosine kinase inhibitors [41], respectively. While EGFR is one of the most important oncogenic drivers in glioma, the clinical efficacy of EGFR-targeted therapy has been disappointing [42]. This observation of acquired intracellular domain EGFR mutations may serve as one of the mechanisms accounting for the low efficacy of EGFR-targeted therapies in glioma. Interestingly, one patient showed acquisition of MGMT methylation and mutations across multiple genes including cKIT, PIK3CA and PTPN11. This patient also carried TP53 and PTEN mutations, which are known to cause genomic instability [43] [44]. These results demonstrate that patients at high risk for genetic instability, as identified by an initial NGS profile, should be profiled at recurrence to identify any new, targetable aberrations. The paired tumor analyses carry some caveats: since only one profile was performed on each particular sample, tumor heterogeneity could not be fully addressed. To mitigate this issue, multiple H&E slides were cut and the most representative area of the sample was circled for testing. Additional caveat of the analysis lies in the unavailability of treatment information. Nevertheless, the high probability of biomarker changes support profiling prior to treatment initiation.

Using SSDI as the source of patient survival, our data shows that the mean overall survival of the 310 GBM patients was 500 days (16.7months), comparable to reported results from the RTOG0525 [45] and RTOG0825 [46] trials and in the TCGA database [8], and therefore, representative of the general glioma population. Our analysis confirms patient age as an important prognostic factor, and that within subgroups defined by age, biomarkers are also closely associated with survival. MGMT-methylation is predictive of longer survival in grade IV patients <=70 years old, but is not in patients > 70 years old. Even though patient treatments were not included in our data, since 2007, temozolomide has become the standard-of-care for newly diagnosed, younger GBM patients, and is increasingly used in older patients, though not as commonly [47]. Therefore, our data is consistent with, but does not prove, the predictive value of MGMT-methylation for temozolomide responsiveness. The prognostic effect of IDH1 mutation was not seen likely due to lack of NGS results before early 2013. Additional markers shown to associate with survival in GBM include PR expression, which has been described as a favorable prognosticator in meningiomas [48], but the effect in GBM [49] [50] remains unknown. Recent preclinical study has suggested progesterone with synergistic effect when combined with temozolomide [51]. Our novel finding in GBM warrants further research to elucidate any implications on therapy and survival. TOPO1 overexpression is indicative of poor prognosis in young GBM patients. Based on the potential association of TOPO1 with irinotecan response [52], it is plausible to design prospective randomized trials validating the TOPO1-irinotecan association in glioma. The poor prognostic effect of TS expression has been reported in other cancer types including NSCLC, and is reported in glioma here for the first time [53]. It's important to note that our survival analysis is limited by only having this data for a small proportion of the patients, and that information are patients' treatments, responses, performance and extent of resection is lacking. Public databases including SSDI have been shown to be reliable sources for patient survival and have aided in cancer research; however, with the limitations stated above, the survival analysis shown here remains exploratory and needs to be validated in an independent cohort or a randomized trial.

In conclusion, we have summarized biomarker data from 1035 GBM tumors submitted for tumor profiling for theranostic purposes. While standard chemotherapy options are limited for GBM, our data is of importance for both clinical consideration and for clinical trial design. We have identified distinct biomarker profiles defined by WHO grades and molecular characteristics including TP53 and IDH1 mutations. The demonstration of biomarker changes within the same patient over time suggests the necessity of profiling before treatment is instituted. Our results provide a biomarker database for therapy consideration and clinical trial design. Prospective trials are underway to confirm the clinical merit of this approach.

## MATERIALS AND METHODS

Biomarker evaluation was performed on consecutive glioma samples submitted to a CLIA-certified laboratory (Caris Life Sciences, Phoenix, AZ) between 2009 and June of 2015. A retrospective analysis was performed to identify biomarker characteristics of the complete cohort and subgroups. Relative risks with 95% confidence intervals were calculated for univariate comparisons, and associated p-values were calculated using the Fisher Exact test. A two-tailed p-value < 0.05 was considered statistically significant and Bonferroni correction was used to correct for multiple comparisons. A logistic regression model was used for multivariate analysis.

This retrospective analysis utilized previously collected, de-identified data and was deemed exempt from IRB oversight; consent requirements were waived by Western Regional Review Board, the IRB of record for Caris Life Sciences.

### Multiplatform tumor profiling

Immunohistochemistry (IHC) was performed on formalin-fixed paraffin-embedded (FFPE) tumor samples using automated staining techniques. IHC results were evaluated independently by board-certified pathologists. Results were categorized into positive or negative by defined thresholds specific to each marker based on published clinical literature that associates biomarker status with patient responses to therapeutic agents. The primary antibody clones and thresholds used can be found in Supplementary Material. Fluorescent *in situ* hybridization (FISH) was performed to detect EGFR gene amplification and 1p19q co-deletion. Chromogenic *in situ* hybridization (CISH) or FISH were both used for Her2/neu and cMET gene amplification. Probes and cutoffs can be found in Supplementary Material.

Next-generation sequencing (NGS) was performed on genomic DNA isolated from FFPE tumor tissue using the Illumina MiSeq platform. Specific regions of 47 genes were amplified using the customized Illumina TruSeq Amplicon Cancer Hotspot panel [15]. All variants reported are detected with >99% confidence based on the mutation frequency present and the amplicon coverage. Average depth of coverage is larger than x1500x. Sanger sequencing included selected regions of BRAF, KRAS, c-KIT, EGFR, NRAS, IDH2 and PIK3CA and was performed using M13-linked PCR primers designed to flank and amplify targeted sequences. MGMT methylation testing was performed on extracted DNA by pyrosequencer-based analysis of 5 CpG sites (CpGs 74-78). Samples with ≥7% and <9% methylation were considered to be equivocal results. Fragment analysis of EGFRvIII was performed on RNA extracted from FFPE samples. Two sets of FAM-linked primers were used to PCR-amplify both the wild type and mutant EGFR alleles, and PCR products were visualized using an ABI 3500xl. Signals generated from the wild-type allele were used as an amplification control and samples were considered positive if EGFRvIII was detected at a level that was 5x higher than the average background signal.

### Data extraction from Social Security Death Index (SSDI)

Patient dates-of-death were extracted from public databases, including the Social Security Death Index, by a research intermediary who ensured that protected health information was removed from datasets delivered to the researchers. The research intermediary estimated patient survival times by calculating the difference between date of death and date of diagnosis. To estimate the effects of biomarkers on survival, a Cox proportional-hazards model was used to calculate the hazard ratio (HR).

## SUPPLEMENTARY MATERIAL TABLES AND FIGURES


