# Supplementary material for: Multi-platform molecular profiling of a large cohort of glioblastomas reveals potential therapeutic strategies
Source: Oncotarget. 2016 Feb 25;7(16):21556–69. doi: 10.18632/oncotarget.7722 (PMC5008305; doi:10.18632/oncotarget.7722)
Supplement: Supplementary file 1 [file oncotarget-07-21556-s001.pdf]

Multi-platform molecular profiling of a large cohort of glioblastomas reveals potential therapeutic strategies

Supplementary Material

Supplementary methodology:

Immunohistochemistry primary antibody clones:

Primary antibodies used are as follows: AR (AR27), EGFR (H11), ER (SP1), PDGFR (polyclonal), Her2 (4B5), ERCC1 (8F1), MGMT (MT23.3), PGP (C494), PR (1E2/100), PTEN (6H2.1), RRM1 (polyclonal), SPARC monoclonal (12251), SPARC (polyclonal), TOPO1 (1D6), TOPO2A (3F6) and TS (TS106/4H4B1), cMET(SP44), TUBB3(Polyclonal), TLE3(polyclonal), PD1 (MRQ-22), PD-L1 (130021/SP142), ALK (D5F3), c-KIT (polyclonal CD117).

Fluorescent and chromogenic in-situ hybridization probes and threshold:

FISH was performed on HER-2/neu [HER-2/CEP17 probe], EGFR [EGFR/CEP7 probe], and cMET [cMET/CEP7 probe] and 1p19q [1p36/1q25 and 19p13/19q13]. CISH was also used for Her-2/neu (INFORM HER-2 Dual ISH DNA Probe Cocktail) and cMET (Ventana).

EGFR amplification was defined by the presence of EGFR/CEP7 ratio of ≥ 2, or ≥ 15 EGFR copies per cell in ≥ 10% of analyzed cells. FISH and CISH assays were both used for evaluation of the HER-2/neu and cMET status. HER-2/CEP17 ratio ≥2 was considered amplified. cMET was considered amplified if ≥ 5 copies per tumor cells were detected on average.1p19q fluorescence *in situ* hybridization was performed with Abbott Molecular probes for 1p36/1q25 and 19p13/19q13. A sample was considered to have both 1p and 19q deletion when both ratios of 1p/1q signals and 19q/19p signals is <0.80.

NextGen Sequencing gene list:

NextGen Illumina customized Truseq panel included hotspot regions of 47 genes: ABL1, AKT1, ALK, APC, ATM, BRCA1, BRCA2, BRAF, CDH1, CSF1R, CTNNB1, EGFR, ERBB2, ERBB4, FBXW7, FGFR1, FGFR2, FGFR3, FLT3, GNA11, GNAS, HNF1A, HRAS, IDH1, JAK2, JAK3, KDR (VEGFR2), KIT, KRAS, MET, MLH1, MPL, NOTCH1, NPM1, NRAS, PDGFR, PIK3CA, PTEN, PTPN11, RB1, RET, SMAD4, SMARCB1, SMO, STK11, TP53, and VHL.

<u>Supplementary Table1:</u> IHC and thresholds used. The IHC staining is recorded as the staining intensity (1+, faint staining, 2+, weak to moderate staining, 3+, strong staining) and percent (0-100%).

Immunohistochemistry Biomarker	Threshold (staining intensity (1+, 2+ or 3+) and percentage (0-100%))
TUBB3	<30% or <2+ or ≥2+ and ≥30%
PTEN	=0+ or ≤50% or ≥1+ and >50%
TOPO1	=0+ or <30% or <2+ or ≥2+ and ≥30%
EGFR	=0+ or =1+ and <10% or ≥1+ and ≥10%
TS	=0+ or ≤3+ and <10% or ≥1+ and ≥10%
TOP2A	=0+ or <10% or ≥1+ and ≥10%
RRM1	=0+ or <50% or <2+ or ≥2+ and ≥50%
ERCC1	<2+ or ≤3+ and <10% or =2+ and <50% or ≥3+ and ≥10% or ≥2+ and ≥50%
TLE3	<30% or <2+ or ≥2+ and ≥30%
PDGFR	<30% or <2+ or ≥2+ and ≥30%
SPARC	<30% or <2+ or ≥2+ and ≥30%
PR	=0+ or <10% or ≥1+ and ≥10%
PGP	=0+ or <10% or ≥1+ and ≥10%
MGMT	=0+ or ≤35% or ≥1+ and >35%
AR	=0+ or <10% or ≥1+ and ≥10%
cMET	<50% or <2+ or ≥2+ and ≥50%
ER	=0+ or <10% or ≥1+ and ≥10%
Her2	≤1+ or =2+ and ≤10% or ≥3+ and >10%
PD-1	1 TIL Count/HPF w/40X Objective; =0+ or ≥1+
PD-L1	<5% or <2+ or ≥2+ and ≥5%
c-KIT	<30% or <2+ or ≥2+ and ≥30%
ALK	<3+ or ≥3+ and ≥1%

Supplementary table 2:

Frequency and types of mutations (protein changes) detected in GBM cohort. P: pathogenic; PP: presumed pathogenic, VUS: variant of unknown significance.

Gene	Protein Changes	Exon	Result	N	Gene	Protein Changes	Exon	Result	N
ABL1	K247R	4	VUS	4	BRCA2	A2717V, D1864N, G2044fs, R2502H, S1632fs, T3033fs, T64N	3, 11, 15, 18, 23	P	1
	R307W	6	VUS	1		T630I	10	VUS	1
	E409	7	VUS	1	cKIT	R271H, N400S	5,7	VUS	1
AKT	D46E	3	VUS	1		P518S	10	VUS	1
	R48C	3	VUS	1		V530I	10	VUS	1
APC	A1358V	16	VUS	1		V530I	10	VUS	1
	E1309fs	16	P	1		E583K	11	VUS	1
	E1317Q	16	VUS	7		E583X	11	VUS	1
	H1375R	16	VUS	1		I571M	11	VUS	1
	H913R, E1317Q	16	VUS	1		M552T	11	VUS	1
	I1307K	16	PP	3		H697Y	14	VUS	1
	L1129S	16	VUS	3		K704N	14	VUS	1
	S1126R	16	VUS	1		L702P	14	VUS	1
	S1144R	16	VUS	1	cMET	E168D	2	VUS	2
	S1340F	16	VUS	1		S186P	2	VUS	1
	S1507N	16	VUS	1		V378I	2	VUS	2
ATM	V410A	9	PP	4		T1010I	14	VUS	4
	P604S	12	VUS	5		D1249N	19	VUS	1
	A1309T	26	VUS	2	CSF1R	A960T	22	VUS	1
	N1719S	34	VUS	1		S939I	22	VUS	1
	S1691R	34	VUS	6	CTNNB1	P16S	3	VUS	1
	K1964E	39	VUS	1	EGFR	D46Y	2	VUS	1
	K2756X	56	P	1		R108K	3	PP	4
BRAF	G460E	11	VUS	1		A289D	7	VUS	3
	G464V	11	P	1		A289T	7	PP	5
	G469A	11	P	1		A289V	7	PP	12
	D594G	15	PP	1		A613T	15	VUS	1
	N581I	15	VUS	1		C620Y	15	PP	1
	V600E	15	P	11		E602K, E608C	15	VUS	1
BRCA1	E23fs	2	P	1		G598V	15	P	6
	I591T	10	VUS	1		K609R	15	VUS	1
	P1238S	14	VUS	1		L619R	15	VUS	1
	G552D	10	VUS	1		E711Q, G719A	18	P	1
	I1275V	14	VUS	1		G719D	18	P	1
	R1347G	14	VUS	2		G724D	28	VUS	1
	S741F	14	VUS	1		N771_P772insH	20	P	1
	I562M	10	VUS	1		N771dup	20	P	1
BRCA2	V2969M	22	VUS	2		R776H	20	P	1
	D777N, E409K	10, 11	VUS	1		S768I	20	P	1
	K1286del, V2138F	11	VUS	1		T790M	20	P	1
	A938fs	11	P	1		V774M	20	VUS	4
	F221L	8	VUS	1		V774M, G598V	20	P	1
	E984K, G2743D, S2216F	11, 18	VUS	1		L858M	21	VUS	1
	R2108C	11	VUS	1	ERBB4	R106H	1	VUS	1
	K1453R	11	VUS	1		E276K, E934K	3	VUS	1
	T3371A	27	VUS	1		P594S	7, 23	VUS	1

Gene	Protein Changes	Exon	Result	N	Gene	Protein Changes	Exon	Result	N
FBXW7	L376fs	8	P	1	PIK3CA	R115L	1	PP	1
	I434T	9	VUS	1		R88Q	1	PP	2
	R465H	9	P	1		R93Q	1	VUS	1
	T456N	9	VUS	1		R93W	1	PP	2
FGFR2	D521G	12	VUS	1		N345I	4	VUS	1
	H544R	12	VUS	1		N345K	4	PP	1
FLT3	S446L	11	VUS	1		C420R	7	PP	1
	S963L	24	VUS	1		D434N	7	VUS	1
GNA11	R202W	4	VUS	1		D549A	9	VUS	1
	P298L	7	VUS	1		E542A	9	P	2
GNAS	Q213H	8	VUS	1		E542K	9	P	4
HNF1A	P291fs	4	P	3		E542V	9	P	1
IDH1	R132C	4	P	1		E545A	9	P	2
	R132G	4	P	3		E545K	9	P	5
	R132H	4	P	39		Q546L	9	P	1
	R132S	4	P	1		Q546R	9	P	2
IDH2	R172G	4	P	1		E726K	13	PP	1
JAK3	V718L	16	VUS	1		G1049R	20	P	1
	V722I	16	VUS	11		H1046R	20	P	1
KDR	Q970H	21	VUS	1		H1047Q	20	P	1
	I1176S	27	VUS	1		H1047R	20	P	7
	I1180L	27	VUS	1		H1047Y	20	P	1
KRAS	G12C	2	P	2		H1048R, M1043I	20	P	1
	G12D	2	P	6		M1043T	20	P	1
	G12V	2	P	1		M1043V	20	P	2
	G13D	2	P	1		T1025A	20	P	1
	V8A	2	VUS	1	PTEN	c.77_79+3delinsG	1	VUS	1
	V112I	4	VUS	1		D24fs	1	P	1
	I163V	5	VUS	1		E18fs	1	P	1
MLH1	S406N	12	VUS	1		L23F	1	VUS	1
NOTCH1	T970A	18	VUS	1		L25F	1	VUS	2
	R1586C	26	VUS	1		R11X	1	P	2
NRAS	G12C	2	P	2		R14del, Y16F	1	VUS	2
	Q61L	3	P	3		R15S	1	VUS	1
	Q61R	3	P	2		S10del	1	VUS	1
PDGFRA	D576G	12	VUS	1		S10R	1	VUS	2
	Y582H	12	VUS	1		T26fs	1	P	1
	N659K	14	P	1		V9D	1	VUS	1
	D842V	18	P	2		Y16_G20del	1	VUS	1
	D846G	18	VUS	1		Y16F	1	VUS	1
	R841_I843del	18	VUS	1		Y27N	1	PP	1
PIK3CA	C90Y	1	VUS	1		c.209_209+1del	3	P	3
	E109del	1	VUS	1		c.209+1_209+4del4	3	P	1
	P104L	1	VUS	1		I67R	3	VUS	1
	R108H	1	PP	1		Y68N	3	PP	1

Gene	Protein Changes	Exon	Result	N	Gene	Protein Changes	Exon	Result	N
PTEN	I203fs	6	P	1	PTPN11	T507K	13	PP	2
	L182_L186delinsVKNHV	6	VUS	1	RB1	E137D	4	VUS	1
	L182V, L186V	6	PP	3		R150X	4	P	1
	L182X	6	P	1		R358X	11	P	2
	M198del	6	VUS	3		W563R	17	PP	1
	M199del	6	PP	1		S576fs	18	P	1
	N184fs	6	P	1		R661W	20	P	1
	P204A	6	VUS	1		R698M	20	VUS	1
	Q171R	6	VUS	1		V714fs	21	P	1
	Q171X	6	P	1		E748fs	22	P	1
	R173G	6	VUS	1	RET	Y791F	13	VUS	4
	S170N	6	PP	1	SMAD4	P139S	3	VUS	1
	Y174C	6	VUS	1		P170S	5	VUS	1
	Y178D	6	VUS	1		S196N	5	VUS	1
	Y180X	6	P	1		Q249P	6	VUS	1
	D252G	7	VUS	1		W509X	12	P	1
	E235D	7	VUS	1	SMARCB1	T72fs	2	P	1
	F241_E242delinsL	7	VUS	1	SMO	R199Q	3	VUS	1
	I253fs	7	P	1		I408V	6	VUS	1
	I253S	7	VUS	1		P641A	11	VUS	1
	K254fs	7	P	1	STK11	R39C	1	VUS	1
	P213fs	7	P	1		F354L	8	VUS	4
	P244_L247del	7	VUS	1	TP53	A159P, C242F	5,7	P	1
	P246L	7	P	1		C135G	5	PP	1
	P248fs	7	P	1		C141G	5	VUS	1
	R233X	7	P	4		C176G	5	P	1
	R173C, c.1026+2T>C	8	P	1		C229fs	7	P	1
	D312fs	8	P	1		C275Y	8	PP	1
	D324fs	8	P	1		C277Y	8	PP	1
	E299X	8	P	1		D281E	8	PP	1
	E307fs	8	P	1		D281G	8	PP	1
	F341fs	8	P	1		E171fs, R175H	5	P	1
	L320fs	8	P	1		E180K	5	PP	1
	N323fs	8	P	1		E204X	6	P	1
	R335X	8	P	3		E271K	8	PP	1
	S287X	8	P	1		E285K	8	PP	2
	T319fs	8	P	4		E285V	8	PP	1
	Y315X	8	P	1		E286K	8	PP	1
PTPN11	A72T	3	PP	2		F109fs	4	P	1
	A72V	3	VUS	1		F134L	5	PP	1
	D61V	3	P	1		F270S	8	PP	1
	D61Y	3	P	1		G154S, R273C	5, 8	P	1
	E76K	3	P	2		G244D	7	PP	1
	Q79R	3	VUS	1		G244S	7	P	3
	T73I	3	PP	1		G245D	7	P	1
	G503V	13	VUS	2		G245I	7	P	1

Gene	Protein Changes	Exon	Result	N	Gene	Protein Changes	Exon	Result	N
TP53	G245S	7	P	1	TP53	R248Q, P278S	7, 8	P	1
	G293W	8	VUS	1		R248W	7	P	5
	H178_S183del	5	P	1		R249S	7	P	1
	H178D	5	PP	1		R267Q	8	PP	2
	H179Q, C275R	5, 8	P	1		R273C	8	P	10
	H179Y	5	P	1		R273C, R306X	8	P	1
	H179Y, R282W	5, 8	P	1		R273H	8	P	9
	H179Y, R342X	5, 10	P	1		R273H, R273C	8	P	1
	H214R	6	PP	1		R273H, V274H	8	P	1
	I195T	6	PP	2		R280G	8	PP	2
	I195T, C242G, R342X	6, 7, 10	P	1		R280I	8	PP	1
	K139N, A276P	5,8	PP	1		R282W	8	P	3
	L111M	4	VUS	1		R306X	8	P	1
	L130V, R273H	5, 8	P	1		R342X	10	P	4
	L145R	5	PP	1		S215G	6	PP	1
	M237V	7	PP	1		S215N	6	PP	1
	M246I	7	PP	1		S227P, R248W	7	P	1
	M340fs	10	P	1		S241F	7	PP	1
	P151A	5	PP	2		S96fs	4	P	1
	P152A, P153S, A159fs	5	P	1		T155I	5	PP	1
	P152L	5	PP	3		T211fs	6	P	1
	P177H	5	PP	1		T230P, T231_I232delinsS	7	PP	1
	P177L	5	PP	1		V143M	5	PP	3
	P190L	6	PP	1		V157F, H179R	5	P	1
	P219L	6	VUS	1		V173G	5	VUS	1
	P223L	6	VUS	1		V173L	5	PP	1
	P278S	8	PP	1		V173M	5	PP	4
	P98fs	4	P	1		V197M	6	PP	1
	P98S	4	VUS	1		V203M	6	VUS	1
	Q136E, V143M	5	PP	1		V216M	6	PP	1
	Q144X	5	P	1		V218del	6	PP	1
	Q167X	5	P	1		V272M	8	PP	1
	R110C	4	PP	1		W146X, R181S	5	P	1
	R158G	5	PP	1		W91X	4	P	2
	R158H	5	P	1		Y126N	5	PP	1
	R158H, R175H	5	P	1		Y163C	5	PP	3
	R158H, R273H	5, 8	P	1		Y205C	6	PP	1
	R175H	5	P	8		Y220C	6	P	2
	R175H, E285K	5, 8	P	1		Y220C, R248Q	6,7	P	1
	R175H, V216M	5,6	P	1		Y220N	6	P	1
	R181H	5	PP	1		Y234C	7	PP	2
	R196Q	6	VUS	1	VHL	V130I	2	VUS	1
	R209X	6	P	1		D197N	3	VUS	1
	R213X	6	P	2		R200W	3	P	1
	R248Q	7	P	5		Y185C	3	VUS	1

Supplementary Table 3: Biomarker-therapy associations of selected markers.

IHC	Associated Therapeutic agents	Association
TUBB3	paclitaxel, docetaxel	Lack of Benefit
PTEN	everolimus, temsirolimus	Benefit
TOPO1	irinotecan, topotecan	Benefit
TS	fluorouracil, capecitabine	Lack of benefit
TOP2A	anthracyclines, etoposide	Benefit
RRM1	gemcitabine	Lack of benefit
ERCC1	cisplatin, carboplatin	Lack of benefit
TLE3	paclitaxel, docetaxel	Benefit
SPARC	nab-paclitaxcel	Benefit
PR	hormonal therapies	Benefit
MGMT	temozolomide	Lack of benefit
cMET	cMET inhibitors	Benefit
ER	hormonal therapies	Benefit
Her2	trastuzumab, lapatinib	Benefit
PD-1	Immune-modulatory agents	Benefit
PD-L1	Immune-modulatory agents	Benefit
**FISH**	**Associated Therapeutic agents**	**Association**
cMET	cMET inhibitors	Benefit
Her2	trastuzumab, lapatinib	Benefit
EGFR	EGFR-targeted therapy	Benefit
1p19q	Procarbazine, vincristine, lomustine	Benefit
**Other**	**Associated Therapeutic agents**	**Association**
EGFR vIII	EGFRvIII-targeted therapy	Benefit
MGMT methylation	temozolomide	Benefit

Grade IV patients <=70 years old
n=251

Marker	HR	Lower CI	Upper CI	p−value
SEQ.MGMT.Me	0.44	0.22	0.87	0.02
IHC.PR	0.61	0.4	0.94	0.02
IHC.TOPO1	1.34	0.99	1.8	0.05
IHC.SPARC	0.75	0.54	1.02	0.07
ISH.cMET	2.66	0.81	8.71	0.11
IHC.TS	1.33	0.94	1.87	0.11
IHC.PTEN	1.26	0.93	1.71	0.13
IHC.TLE3	0.72	0.47	1.11	0.14
NGS.PTEN	0.35	0.08	1.52	0.16
IHC.TUBB3	0.5	0.18	1.37	0.18
IHC.Ki67	2.45	0.56	10.79	0.24
KRAS	1.96	0.61	6.32	0.26
IHC.TOP2A	1.18	0.85	1.63	0.32
NGS.TP53	0.68	0.32	1.49	0.34
ISH.cMYC	0.51	0.11	2.29	0.38
ISH.EGFR	1.16	0.83	1.63	0.38
IHC.AR	0.75	0.31	1.84	0.53
IHC.MRP1	1.11	0.76	1.63	0.59
IHC.ERCC1	0.94	0.68	1.29	0.69
IHC.EGFR	1.13	0.59	2.18	0.71
IHC.MGMT	1.11	0.61	2.01	0.74
PIK3CA	1.14	0.49	2.67	0.76
IHC.p53	0.88	0.34	2.24	0.78
IHC.PGP	0.95	0.58	1.54	0.83
IHC.BCRP	1.07	0.58	1.95	0.83
BRAF	1.1	0.35	3.49	0.87
IHC.COX.2	0.92	0.32	2.66	0.87
IHC.PDGFR	0.98	0.53	1.82	0.96
IHC.RRM1	1.01	0.74	1.36	0.96

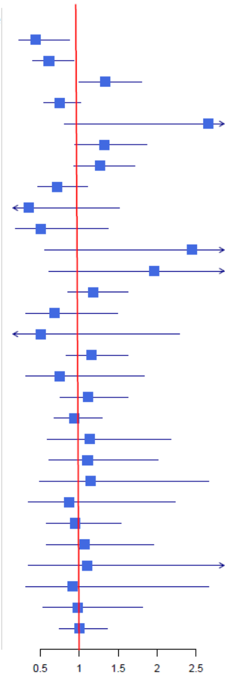

<u>Supplementary Figure 1:</u> Forest plot showing association of biomarkers and patient survival in GBM patients <=70 years old.

Grade IV patients >70 years old
n=59

Marker	HR	Lower CI	Upper CI	p−value
IHC.PGP	0.31	0.12	0.81	0.02
IHC.PR	0.23	0.07	0.8	0.02
IHC.TS	2.61	1.11	6.16	0.03
IHC.BCRP	0.14	0.02	1.22	0.07
IHC.TUBB3	5.48	0.66	45.65	0.12
IHC.PTEN	1.97	0.77	5.03	0.16
IHC.PDGFR	0.45	0.1	2.07	0.3
IHC.MRP1	0.67	0.31	1.48	0.32
IHC.cMET	0.52	0.14	1.92	0.33
IHC.RRM1	0.74	0.39	1.41	0.36
IHC.ERCC1	0.74	0.36	1.53	0.42
SEQ.MGMT.Me	0.66	0.24	1.81	0.42
IHC.SPARC	0.79	0.42	1.48	0.46
IHC.MGMT	1.39	0.42	4.62	0.59
ISH.EGFR	0.85	0.39	1.88	0.7
IHC.TOP2A	0.89	0.47	1.67	0.71
IHC.AR	1.18	0.42	3.32	0.76
IHC.TOPO1	0.91	0.5	1.67	0.77
IHC.TLE3	0.94	0.33	2.7	0.91

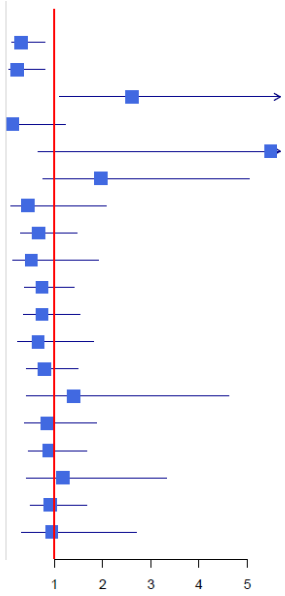

<u>Supplementary Figure 2:</u> Forest plot showing association of biomarkers and patient survival in GBM patients >70 years old.
